# Phenotypic characterisation of breast cancer: the role of CDC42 

**DOI:** 10.1007/s10549-017-4267-8

**Published:** 2017-04-27

**Authors:** Eleni Chrysanthou, Kylie L. Gorringe, Chitra Joseph, Madeleine Craze, Christopher C. Nolan, Maria Diez-Rodriguez, Andrew R. Green, Emad A. Rakha, Ian O. Ellis, Abhik Mukherjee

**Affiliations:** 1 Division of Cancer and Stem Cells, School of Medicine, University of Nottingham and Nottingham University Hospitals NHS Trust, City Hospital Campus, Nottingham, NG5 1PB UK; 2 Cancer Genomics Program, Peter MacCallum Cancer Centre, Melbourne, Australia; 3 The Sir Peter MacCallum Department of Oncology, University of Melbourne, Parkville, Australia

**Keywords:** CDC42, Immunohistochemistry, Luminal breast cancer, Prognosis

## Abstract

**Purpose:**

The molecular landscape of breast cancer (BC), especially of the Luminal A subtype, remains to be fully delineated. Transcriptomic data show that Luminal A tumours are enriched for aberrant expression of genes in the cell division control 42 homolog (CDC42) pathway. This study aims to investigate the protein expression of CDC42 in BC and assess its clinicopathological significance.

**Methods:**

Expression of CDC42 protein was examined by immunohistochemistry on tissue microarrays in a well-characterised cohort of 895 early-stage (I–IIIa) primary invasive BCs.

**Results:**

CDC42 expression was observed in both the cytoplasm and the nucleus of BC cells. High nuclear CDC42 expression demonstrated a significant correlation with ER-positive, low-grade tumours and was more common in the lobular histological subtype (all *p* < 0.001). In contrast, cytoplasmic CDC42 showed increased expression in the ductal subtype (*p* < 0.001) and correlated with negative prognostic features such as larger size, higher grade (*p* < 0.05) and higher Ki67 labelling index (*p* = 0.001). Nuclear CDC42 expression was associated with a longer BC-specific survival in all cases (*p* = 0.025) and in luminal ER-positive tumours (*p* = 0.011). In multivariate analyses including size, grade, lymph node stage and intrinsic subtype, CDC42 was an independent prognostic factor (*p* = 0.032).

**Conclusion:**

The results indicate that CDC42 is an important molecule in luminal BC, with prognostic significance.

**Electronic supplementary material:**

The online version of this article (doi:10.1007/s10549-017-4267-8) contains supplementary material, which is available to authorized users.

## Introduction

Breast cancer (BC) is a heterogeneous disease with multiple subtypes related to the oestrogen receptor (ER) status, the presence of *ERBB2* amplification and also the genetic and transcriptomic landscape. While large genomic studies have identified subgroups with different clinical outcomes [1–3], the aberrant pathways driven by the various genetic aberrations identified in these subgroups remain to be elucidated. In particular, studies are required to resolve the landscape of the Luminal A intrinsic subtype, the most common molecular subtype of BC. This group of ER-positive tumours are usually low grade and have a good prognosis with a good response to hormone therapy. However, a small subset of Luminal A tumours will progress and clinically recur.

Based on genomic and transcriptomic analysis by the Molecular Taxonomy of the Breast Cancer International Consortium (METABRIC) group, Luminal A tumours are enriched for aberrant expression of genes in the cell division control 42 homolog (CDC42) pathway [2] and linked by low levels of copy number aberration. The protein kinase signalling pathway protein CDC42 is a plasma membrane-associated small GTPase which phases between an active GTP-bound and an inactive GDP-bound state [4]. When CDC42 is in an active state, it binds to a variety of effector proteins to control various cellular procedures such as regulation of the actin cytoskeleton, cell migration and progression through G1 phase of the cell cycle to enter S phase for DNA synthesis.

CDC42 is expressed at low levels in normal breast tissue and elevated in breast carcinomas [5], with an essential role in normal mammary development [6]. Despite the importance of the Rho-GTPase pathway in BC, CDC42 protein expression has not been evaluated in a large cohort of BCs with clinical outcome data. This study was thus conducted to investigate the role of CDC42 protein in invasive BCs including correlations with other BC-related biomarkers, clinicopathological variables and disease outcome.

## Materials and methods

### Study cohort

This study was conducted on the well-characterised Nottingham Tenovus Primary Breast Carcinoma series (*n* = 1048), which includes patients at Nottingham City Hospital between 1990 and 1998. The study was approved by the Nottingham Research Ethics Committee 2. Patients were under the age of 70 years and managed in a uniform manner [7]. Clinicopathological parameters recorded include histological tumour type, tumour size, grade and axillary lymph node stage. The series is also annotated with an immunohistochemical repository of a wide range of biomarkers including hormone receptors [oestrogen receptor (ER), progesterone receptor (PgR)], epidermal growth factor receptor family (EGFR and HER2), cytokeratins (basal cytokeratin: CK5/6), the proliferation marker Ki67 and E-cadherin [7]. Survival data were analysed prospectively with disease-specific survival (DSS) defined as the interval in months from primary surgery to patient death caused by BC.

### Western blotting

For validation of CDC42 antibody specificity, Western blotting was performed on whole cell lysates of MCF-7, SKBr3 and MDA-MB231 human breast cancer cell lines (obtained from the American Type Culture Collection; Rockville, MD, USA) using CDC42 antibody (clone PA1-092) at 1:1000 dilution and fluorescent secondary antibodies at 1:15,000 were used (IR Dye 800CW donkey anti-rabbit and 680RD donkey anti-mouse, LI-COR Biosciences, UK). 5% milk (Marvel original dried skimmed milk, Premier Food Groups Ltd, St Albans, UK) was used for blocking. Mouse β-Actin (A5441, Sigma-Aldrich; Clone AC-15; Sigma, UK) at 1:5000 was used as a house-keeping protein. A protein ladder (PageRuler Plus Prestained Protein Ladder, ThermoScientific, Waltham, MA, USA) was included. The fluorescence was then detected using the LI-COR Odyssey Fc machine to visualise the bands, with wavelengths 600, 700 and 800.

### Immunohistochemistry

Expression of the CDC42 protein in BC was assessed by immunohistochemistry (IHC), using the Novocastra Novolink polymer detection system (Leica, Newcastle, UK). In brief, BC tissue microarray (TMA) sections were deparaffinised with xylene and rehydrated through 100% ethanol. Heat-induced retrieval of antigen epitopes was performed in citrate solution (pH 6.0). TMAs were stained with CDC42 antibody (clone PA1-092, 1:30) for 30 min. 3,3′-Diaminobenzidine tetrahydrochloride (Novolink DAB substrate buffer plus) was used as a chromogenic substance. TMA sections were counterstained with haematoxylin for 6 min. Human tonsil sections were used as a positive control while a negative control was achieved by omitting the application of the primary antibody.

### Immunohistochemical scoring

Stained TMAs were scored using the semi-quantitative H-score (Histochemical score) visual approach taking into consideration the intensity of staining and the percentage of stained cells within each tissue core [8]. Both nuclear and cytoplasmic staining were scored separately: staining intensity was scored as 0, 1, 2 or 3 for negative, weak, moderate and strong, respectively. Final scores were obtained by multiplying each staining intensity by its proportion, summed up as an H-score ranging from 0 to 300. All cases were scored blinded to clinicopathological and outcome data. TMAs were double scored for inter-observer variation.

### Analysis of external datasets

Publically available normalised gene expression (RNAseq) and protein mass spectrometry data, as well as clinicopathologic information, were downloaded from the Cancer Genome Atlas (TCGA, [1]) data portal. Gene expression and clinicopathologic information were also obtained from METABRIC collaborators [2]. All analyses were performed in the program R. The results published here are, in part, based on data generated by TCGA project established by the NCI and NHGRI. Information about TCGA and the investigators and institutions who constitute the TCGA research network can be found at http://cancergenome.nih.gov.

### Statistical analysis

Three groups were used for correlation analyses—negative (H-score = 0), low (H-score 10–150) and high (H-score > 150). Statistical analyses were performed in the program R. Correlations were assessed using the Chi-square test (*χ* ^2^ test). Cox regression analysis was performed for survival analysis (coxph), reporting the log-rank test. StepAIC was used for Akaike information criterion modelling. A *p* value of <0.05 (two-sided) was considered to be statistically significant.

## Results

### Analysis of METABRIC and TCGA data

Genomic profiling of BC by the METABRIC consortium encompassing gene expression and copy number data identified ten molecular subtypes called “integrative clusters” (ICs) [2]. The Luminal A type tumours were seen to group in clusters IC3, IC4, IC7 and IC8. We noted that CDC42 signalling was a highly ranked pathway in the IC4 group in the METABRIC study, and was also positively associated with IC3. In contrast, this pathway scored a zero in the other Luminal A-dominated clusters IC7 and IC8. We hypothesised that CDC42 signalling could be useful in delineating a subgroup of Luminal A tumours with different phenotypic characteristics. We focused on the central signalling protein in this pathway, CDC42. Further exploration of the genomics data in the METABRIC cohort found that while *CDC42* itself was differentially expressed between integrative clusters, the highest mRNA expression was not found in either IC3 or IC4 (Supplementary Fig. 1A). However, analysis of The Cancer Genome Atlas (TCGA) mRNA and protein mass spectrometry data showed that *CDC42* mRNA and protein were poorly correlated (Spearman *r* = 0.16, *p* = 0.09, Supplementary Fig. 1B). Thus, *CDC42* mRNA alone may not be a good proxy for CDC42 protein expression or pathway activation.

### Expression of CDC42 by immunohistochemistry

**Fig. 1:**
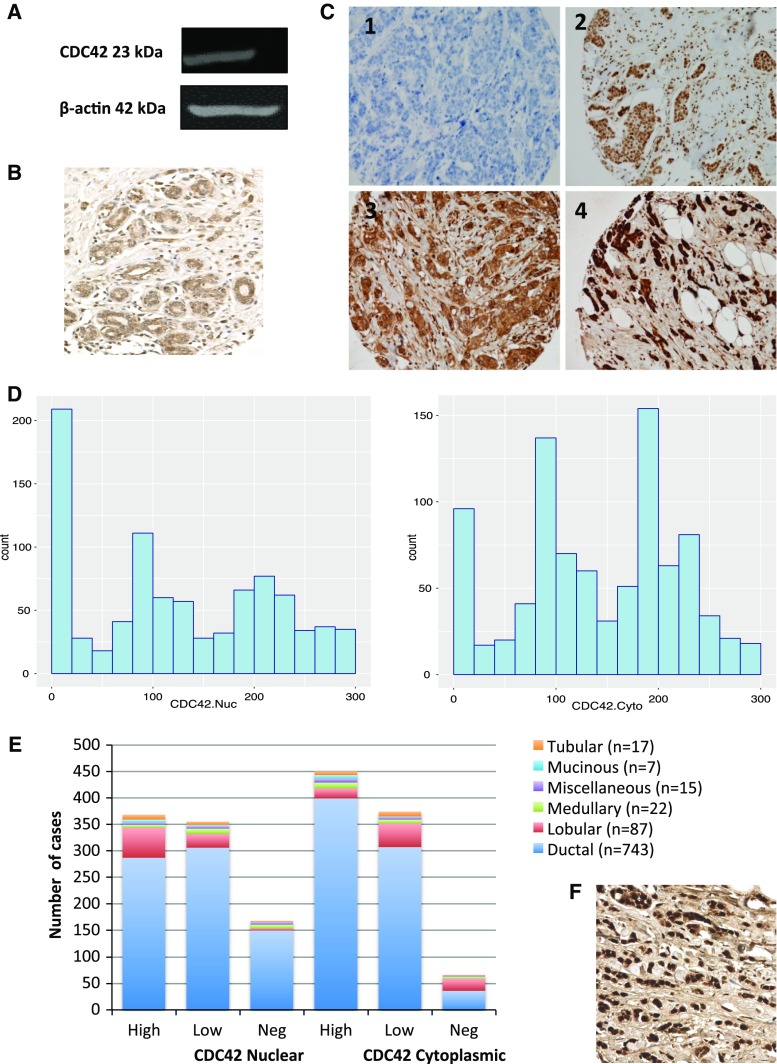
Expression of CDC42 in breast cancer. **a** Western Blotting analysis using anti-CDC42 Polyclonal Antibody (PA1-092) showing band at the expected size (23 kDa). **b** CDC42 expression in terminal duct lobular units using IHC. **c** CDC42 expression in TMA cores using IHC. Intensity levels of staining are shown: *1* negative, *2* weak, *3* moderate and *4* strong expression (×20 magnification). **d** Histograms of H-scores for nuclear and cytoplasmic staining. **e** Distribution of cases across different histological subtypes, showing the increased proportion of lobular cases with high CDC42 nuclear staining but low or negative cytoplasmic staining. **f** Example of lobular carcinoma showing strong nuclear staining

CDC42 showed both nuclear and cytoplasmic staining in the invasive tumour cells (Fig. 1a). Nuclear and cytoplasmic expression were positively correlated (Spearman *r* = 0.38, *p* < 0.001). However, for those cases that were part of the METABRIC study (*n* = 143), there was no correlation between IHC H-score and mRNA expression by microarray, consistent with TCGA mRNA/mass spectrometry data (nuclear staining Spearman *r* = −0.097, *p* = 0.25; cytoplasmic staining *r* = −0.12, *p* = 0.14, Supplementary Fig. 1C). The specificity of the antibody was validated by Western blotting analysis, which showed a single specific band at the predicted size (23 kDa) (Fig. 1b).

The distribution of H-scores from the 895 successfully scored cases suggested tri-modality for both nuclear and cytoplasmic staining, with peaks at 0, 100 and 220 for nuclear, and 0, 100 and 200 for cytoplasmic (Fig. 1c). Three groups were therefore evaluated for correlation with phenotypic tumour features—negative (H-score = 0), low (H-score 10–150) and high (H-score > 150).

### Correlations with clinicopathological parameters

**Fig. 2:**
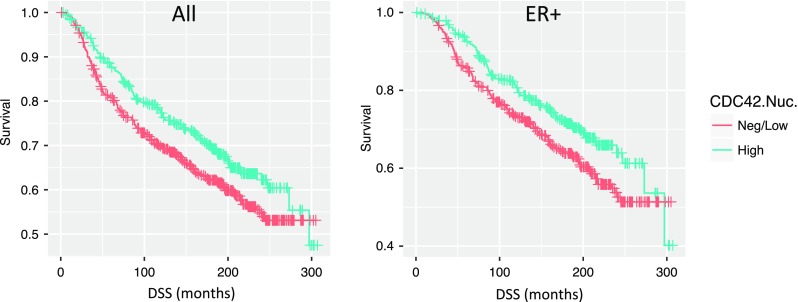
Kaplan–Meier plots for CDC42 nuclear expression: in all cases, ER-positive cases and ER-positive/HER2-negative cases

Nuclear CDC42 expression showed significant negative associations with tumour grade (*p* < 0.001), tumour size (*p* < 0.001) and HER2 status (*p* = 0.018) but a positive correlation with ER status (*p* < 0.001) (Tables 1, 2). Histological subtype was also significantly associated with CDC42 nuclear staining (*p* < 0.001, Fig. 2): lobular types had a higher proportion of cases with high nuclear expression of CDC42 (65.5%), compared to ductal types (38.6% high expression). Thus, high nuclear expression of CDC42 was strongly associated with tumours carrying good prognostic features such as low grade, non-ductal histology, ER positivity, HER2 negativity and smaller size. In contrast, high cytoplasmic CDC42 expression is more common in cases with a ductal histology (53.8%) than lobular (21.8%). CDC42 cytoplasmic staining also showed correlations with tumour size (*p* = 0.04) and grade (*p* = 0.014).

**Table 1:** Correlation of CDC42 protein expression with clinicopathological parameters

	Nuclear				Cytoplasmic			
Feature	Negative	Low	High	*p* value (*χ* ^2^)	Negative	Low	High	*p* value (*χ* ^2^)
Age (years)								
≥50	110 (19.2%)	231 (40.4%)	231 (40.4%)	0.70 (0.702)	48 (8.4%)	241 (42.1%)	283 (49.5%)	0.33 (2.23)
<50	56 (17.8%)	123 (39.0%)	136 (43.2%)		18 (5.7%)	132 (42.0%)	164 (52.2%)	
Size (mm)								
≥20	105 (22.5%)	198 (42.5%)	163 (35.0%)	**<0.001** (18.1)	40 (8.6%)	208 (44.7%)	217 (46.7%)	**0.039** (6.47)
<20	63 (14.8%)	157 (36.9%)	205 (48.2%)		26 (6.1%)	166 (39.1%)	233 (54.8%)	
Grade								
1	24 (19.4%)	45 (36.3%)	55 (44.4%)	**<0.001** (28.8)	8 (6.5%)	54 (43.5%)	62 (50.0%)	**0.014** (12.5)
2	36 (12.0%)	109 (36.3%)	155 (51.7%)		33 (11.0%)	134 (44.7%)	133 (44.3%)	
3	107 (23.1%)	200 (43.1%)	157 (33.8%)		25 (5.4%)	186 (40.2%)	252 (54.4%)	
Lymph node stage								
1	115 (21.1%)	214 (39.3%)	216 (39.6%)	0.24 (5.48)	44 (8.1%)	235 (43.2%)	265 (48.7%)	0.29 (5.004)
2	43 (15.8%)	111 (40.8%)	118 (43.4%)		14 (5.1%)	112 (41.2%)	146 (53.7%)	
3	9 (12.7%)	29 (40.8%)	33 (46.5%)		8 (11.3%)	27 (38.0%)	36 (50.7%)	
Histological type								
Ductal	150 (20.2%)	306 (41.2%)	287 (38.6%)	**<0.001** (36.4)	36 (4.9%)	307 (41.4%)	399 (53.8%)	**p** **<** **0.001** (76.1)
Lobular	4 (4.6%)	26 (29.9%)	57 (65.5%)		23 (26.4%)	45 (51.7%)	19 (21.8%)	
Medullary	7 (31.8%)	10 (45.5%)	5 (22.7%)		4 (18.2%)	7 (31.8%)	11 (50.0%)	
Mucinous	0 (0.0%)	2 (28.6%)	5 (71.4%)		0 (0.0%)	1 (14.3%)	6 (85.7%)	
Tubular	2 (11.8%)	6 (35.3%)	9 (52.9%)		1 (5.9%)	9 (52.9%)	7 (41.2%)	
Other	5 (33.3%)	5 (33.3%)	5 (33.3%)		2 (13.3%)	5 (33.3%)	8 (53.3%)	

Significant *p* values are represented in bold

**Table 2:** Correlation of CDC42 protein expression with other biomarkers

	Nuclear				Cytoplasmic			
Feature	Negative	Low	High	*p* value (*χ* ^2^)	Negative	Low	High	*p* value (*χ* ^2^)
Oestrogen receptor								
Negative	64 (27.7%)	94 (40.7%)	73 (31.6%)	**<0.001** (19.6)	14 (6.1%)	96 (41.6%)	121 (52.4%)	0.59 (1.05)
Positive	104 (15.9%)	259 (39.5%)	292 (44.6%)		52 (8.0%)	276 (42.2%)	326 (49.8%)	
Progesterone receptor								
Negative	87 (24.2%)	149 (41.4%)	124 (34.4%)	**<0.001** (15.8)	24 (6.7%)	166 (46.1%)	170 (47.2%)	0.14 (4.00)
Positive	74 (14.9%)	196 (39.6%)	225 (45.5%)		38 (7.7%)	194 (39.3%)	262 (53.0%)	
HER2 status								
Negative	131 (17.8%)	289 (39.3%)	316 (42.9%)	**0.018** (8.08)	58 (7.9%)	314 (42.7%)	363 (49.4%)	0.28 (2.57)
Positive	31 (25.2%)	55 (44.7%)	37 (30.1%)		7 (5.7%)	46 (37.4%)	70 (56.9%)	
Triple negative								
Non-triple	118 (16.6%)	284 (39.9%)	310 (43.5%)	**<0.001** (16.2)	53 (7.5%)	300 (42.2%)	358 (50.4%)	0.99 (0.029)
Triple	44 (28.8%)	64 (41.8%)	45 (29.4%)		12 (7.8%)	64 (41.8%)	77 (50.3%)	
Intrinsic subtype								
ER−, HER2+	14 (25.0%)	24 (42.9%)	18 (32.1%)	**<0.001** (24.3)	1 (1.8%)	23 (41.1%)	32 (57.1%)	0.083 (11.2)
Luminal A	25 (12.5%)	76 (38.0%)	99 (49.5%)		20 (10.0%)	95 (47.5%)	85 (42.5%)	
Luminal B	58 (16.5%)	148 (42.2%)	145 (41.3%)		22 (6.3%)	137 (39.1%)	191 (54.6%)	
ER−, HER2−	46 (28.6%)	66 (41.0%)	49 (30.4%)		12 (7.5%)	68 (42.2%)	81 (50.3%)	
Ki67								
<10%	32 (13.9%)	88 (38.3%)	110 (47.8%)	**0.035** (6.7)	24 (10.4%)	109 (47.4%)	97 (42.2%)	**0.001** (13.7)
≥10%	101 (20.1%)	206 (41.0%)	195 (38.8%)		25 (5.0%)	202 (40.3%)	274 (54.7%)	
E-cadherin								
Negative/low	59 (18.8%)	134 (42.8%)	120 (38.3%)	0.50 (1.39)	33 (10.6%)	148 (47.4%)	131 (42.0%)	**<0.001** (19.4)
Positive	96 (18.4%)	205 (39.3%)	221 (42.3%)		26 (5.0%)	204 (39.1%)	292 (55.9%)	
EGFR								
Negative	120 (17.4%)	268 (38.8%)	303 (43.8%)	**0.023** (7.52)	57 (8.3%)	285 (41.3%)	348 (50.4%)	0.16 (3.71)
Positive	39 (22.3%)	79 (45.1%)	57 (32.6%)		7 (4.0%)	75 (42.9%)	93 (53.1%)	
CK5/6								
Negative	237 (42.2%)	224 (39.9%)	101 (18.0%)	**0.003** (11.75)	286 (50.9%)	238 (42.3%)	38 (6.8%)	0.443 (1.63)
Positive	29 (25.9%)	52 (46.4%)	31 (27.7%)		59 (52.7%)	49 (43.8%)	4 (3.6%)	

Significant *p* values are represented in bold

We tested for an association of CDC42 with intrinsic subtype using the Gallen IHC system [9], whereby Luminal A tumours are defined as ER+, Ki67-low, Luminal B are ER+ and either Ki67-high or HER2+, HER2 tumours are ER−, HER2+, and Negative tumours are ER−, HER2−. High nuclear CDC42 staining was strongly correlated with the Luminal-type tumours (*p* < 0.001, Table 2). Cases with low CDC42 were significantly more likely to be triple negative for ER, PR and HER2 (28.8% vs 16.6%, *p* = <0.001).

For the subset of cases that were part of the METABRIC cohort (*n* = 144), we tested whether CDC42 IHC H-scores were associated with integrated cluster membership. A statistically significant association of nuclear CDC42 staining with IC subgroup was found (*p* = 0.04, one-way ANOVA, Supplementary Fig. 2). In particular, high CDC42 nuclear staining was most prevalent in the luminal IC groups: IC3 (46% high CDC42), IC4 (50%), IC7 (82%) and IC8 (71%) compared to all others (20%); however, Tukey post-tests were not significant, most likely due to the small number of cases in each group. Cytoplasmic CDC42 staining was not associated with integrative cluster membership (*p* = 0.14).

We compared CDC42 nuclear staining with the expression of other important breast cancer proteins for which IHC data were also available (Table 2). Significant negative associations were observed with Ki67 (*p* = 0.035) and EGFR (*p* = 0.023), and a positive correlation was observed with basal cytokeratin CK5/6 (*p* = 0.003), but not E-cadherin. Cytoplasmic CDC42 showed positive correlations with the expression of Ki67 (*p* = 0.001) and E-cadherin (*p* < 0.001) but not EGFR, or CK5/6.

In TCGA mass spectrometry data, the association of CDC42 protein expression and EGFR protein levels was validated (Spearman *r* = 0.25, *p* = 0.01); however, other associations were of either borderline significance (E-cadherin *r* = 0.19, *p* = 0.059, Ki67 *r* = 0.16, *p* = 0.099) or not significant (CK5/6 *r* = 0.003, *p* = 0.97). This is perhaps not surprising given the different directions of associations seen with nuclear versus cytoplasmic staining by IHC.

### Correlation with patient outcome

In a univariate analysis using the IHC H-scores, high CDC42 nuclear staining was significantly associated with improved disease-specific survival (DSS) (Fig. 2, likelihood ratio test, *p* = 0.025). Similarly, there was a borderline significant result for nuclear CDC42 staining to affect disease-free survival (DFS) (*p* = 0.0885). Because CDC42 nuclear staining was strongly associated with ER-positive status, we also performed a subgroup analysis, stratified by ER status. CDC42 nuclear staining was still associated with DSS in ER-positive cases (*p* = 0.011, Fig. 2), but not ER-negative cases (*p* = 0.65). Similar to the full cohort, CDC42 was associated with DFS with only borderline significance in ER-positive cases (*p* = 0.08). No association with patient DSS or DFS was observed for cytoplasmic CDC42 staining in either the full cohort or by ER status.

Multivariate survival analysis to evaluate the impact of other factors on survival including lymph node stage, grade, tumour size, ER status and HER2 status found that in both the full and ER-positive cohorts, CDC42 nuclear staining was not an independent prognostic factor (*p* = 0.17, *p* = 0.086, respectively, Table 3). However, we also performed Akaike information criterion modelling, including lymph node stage, grade, tumour size, histological subtype and Gallen subtype. Nuclear CDC42 staining was included in the final model for both DSS and DFS, and was individually significant in each model (DSS, *p* = 0.032, DFS, *p* = 0.031, Table 3). Cytoplasmic CDC42 staining was not included in any survival model.

**Table 3:** Multivariate survival analysis

	Hazard ratio	95% confidence interval	*p* value
All (DSS)			
CDC42 nuclear H-score	0.999	0.998–1.00	0.176
Size < 20 mm	0.705	0.57–0.93	**0.005**
Grade	1.582	1.25–1.88	**9.1** **×** **10** ^**−6**^
Lymph node stage	1.762	1.44–2.04	**7.2** **×** **10** ^**−11**^
ER+ status	1.091	0.83–1.43	0.530
HER2+ status	1.555	1.12–2.00	**0.003**
ER positive (DSS)			
CDC42 nuclear H-score	0.999	0.997–1.00	0.086
Size < 20 mm	0.713	0.535–0.95	**0.021**
Grade	1.626	1.315–2.01	**7.2** **×** **10** ^**−6**^
Lymph node stage	1.749	1.423–2.15	**1.1 × 10** ^**−7**^
HER2+ status	1.529	1.051–2.23	**0.027**
Akaike (DSS)			
CDC42 nuclear H-score	0.999	0.997–1.00	**0.032**
Size < 20 mm	0.730	0.562–0.95	**0.018**
Grade	1.379	1.081–1.76	**0.010**
Luminal A	3.902	0.885–17.20	0.072
Luminal B	3.643	0.990–13.41	0.052
ER/HER2 Negative	3.382	0.840–13.62	0.086
Lymph node stage	4.440	2.373–8.31	**3.1 × 10** ^**−6**^
Luminal A: stage	0.269	0.121–0.60	**0.001**
Luminal B: stage	0.409	0.211–0.79	**0.008**
ER/HER2 Negative: stage	0.365	0.176–0.76	**0.007**

Significant *p* values are represented in bold

## Discussion

In the present study, we investigated the expression of CDC42 in a heterogeneous group of patients with invasive BC. CDC42 overexpression has been reported in several other malignancies [10–14], including invasive breast ductal carcinomas [5, 15]. CDC42 was expressed in a higher frequency in the cytoplasm (92.4%) than in the nucleus (80.7%) in our BC cohort, similar to the observations of Halon et al., who found a predominant cytoplasmic localisation of CDC42 [15]. We identified different phenotypic correlations of nuclear versus cytoplasmic expression of CDC42, with high nuclear expression correlating with better prognostic features. This result is consistent with the study performed by Halon et al., where nuclear expression was inversely correlated with lymph node metastasis. However, this earlier study was too small (*n* = 85) to demonstrate a significant survival difference as shown here for nuclear CDC42 expression.

In the cytoplasm, CDC42 acts as a regulator of signal transduction pathways involved in the remodelling of the actin cytoskeleton and regulation of cell polarity [16] and also plays an important role in controlling cell proliferation and stimulating cell cycle progression through G1 phase to S phase via c-Jun [17]. In the present study, we found that higher CDC42 cytoplasmic expression was associated with increased expression of Ki67, whereas the nuclear component showed no correlation to proliferation markers. This result is similar to that seen by Ma et al. [13] in their large (*n* = 339) analysis of breast tumours, although subcellular localisation was not recorded, nor was survival information available. They also observed a positive correlation with TNM stage and lymph node metastasis, which we did not see.

Interestingly, we found an association of CDC42 nuclear expression with special histological tumour types such as lobular and tubular tumours. These tumour types have distinct morphologies e.g. a single cell pattern in the lobular type and tubule formation in the tubular type. As CDC42 is important in cytoskeleton remodelling, it could be involved in contributing to the morphology of these tumours. Indeed, CDC42 overexpression in mouse epithelial mammary cells in vivo leads to hyperbranching of ducts and abnormal terminal end bud morphology [18]. Lobular carcinoma cells are discohesive by nature and loss of E-cadherin is a hallmark of lobular carcinoma. CDC42 GTPase-activating protein (CdGAP) uses its proline-rich domain to form a complex with the epithelial–mesenchymal transition regulator Zeb2 to repress E-cadherin expression [19]. This may be one of the underlying molecular pathways leading to the morphological lobular appearance.

The significance of the presence of CDC42 in the nucleus is unclear, as the literature does not suggest an active role for the protein in this subcellular compartment. However, the related protein Rac1 has been shown to be sequestered in the nucleus for ubiquitin-mediated proteolytic degradation [20], and possibly for an active role related to proliferation [21]. CDC42 is also susceptible to such degradation [22] and contains a conserved C-terminal nuclear localisation signal that could mediate transfer to the nucleus [23]. Therefore, one possibility is that CDC42 is also degraded in the nucleus, and its presence there in breast cancer could represent some deregulation of normal protein turnover. Alternatively, as for Rac1, CDC42 may play a as yet to be determined role in the nucleus that is active in breast cancer cells.

In conclusion, CDC42 seems to be a key determinant of low-grade ER-positive breast cancers with prognostic significance. Subcellular localisation may be important in determining breast cancer morphology and further functional studies in morphological subtypes are warranted.

## Electronic supplementary material

Below is the link to the electronic supplementary material.

Supplementary material 1 (PDF 142 kb)
