# Supplementary material for: Phenotypic characterisation of breast cancer: the role of CDC42
Source: Breast Cancer Res Treat. 2017 Apr 27;164(2):317–25. doi: 10.1007/s10549-017-4267-8 (PMC5487723; doi:10.1007/s10549-017-4267-8)
Supplement: Supplementary file 1 — Supplementary material 1 (PDF 142 kb) [file 10549_2017_4267_MOESM1_ESM.pdf]

Supplementary Figure 1

A

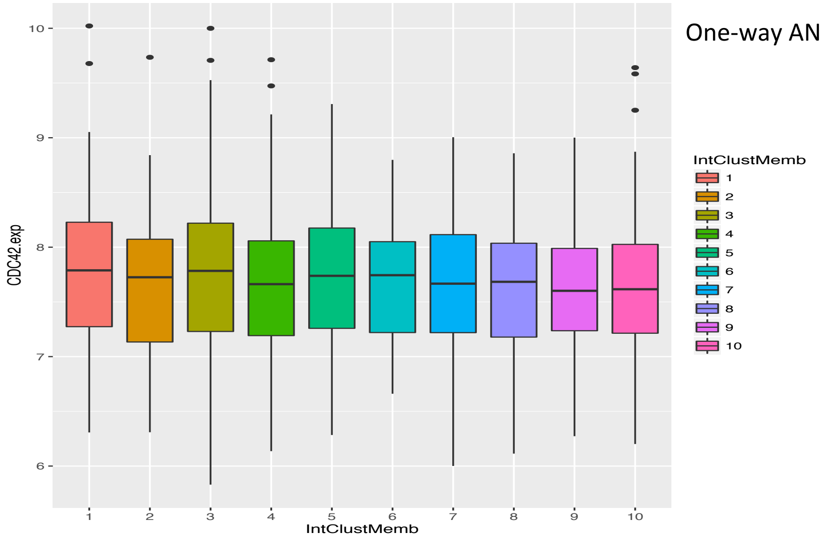

One-way ANOVA p=0.03

IC	CDC42.mean
1	7.761183
2	7.653318
3	7.727817
4	7.614756
5	7.704523
6	7.694870
7	7.631558
8	7.584317
9	7.589938
10	7.593837

B

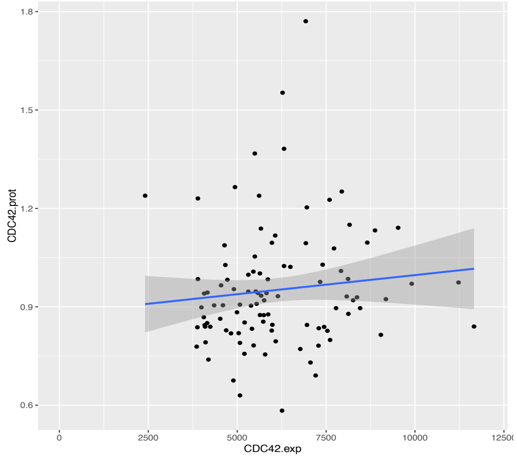

C

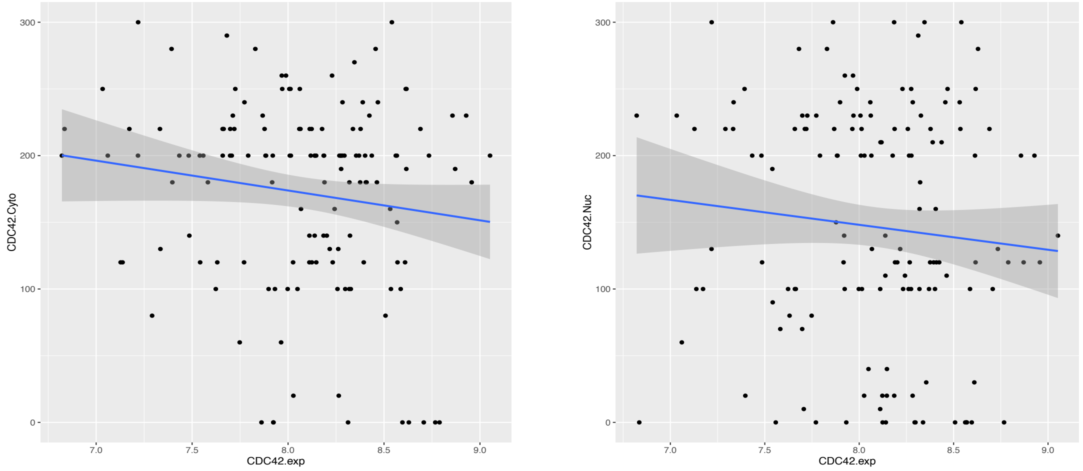

A. mRNA expression of *CDC42* in METABRIC data by integrated cluster membership (IntClustMemb). While the overall expression differed between clusters (p=0.03, one-way ANOVA), none of the Tukey post-tests were statistically significant. Also shown is the mean expression value for each of the ICs
B. In TCGA breast cancer data, *CDC42* mRNA expression (CDC42.exp) is not significantly correlated with mass spectrometry protein levels (Spearman r=0.16, p=0.09).
C. mRNA expression of *CDC42* from METABRIC cases did not correlate with either cytoplasmic (left) or nuclear (right) expression by immunohistochemistry (H-scores)

Supplementary Figure 2

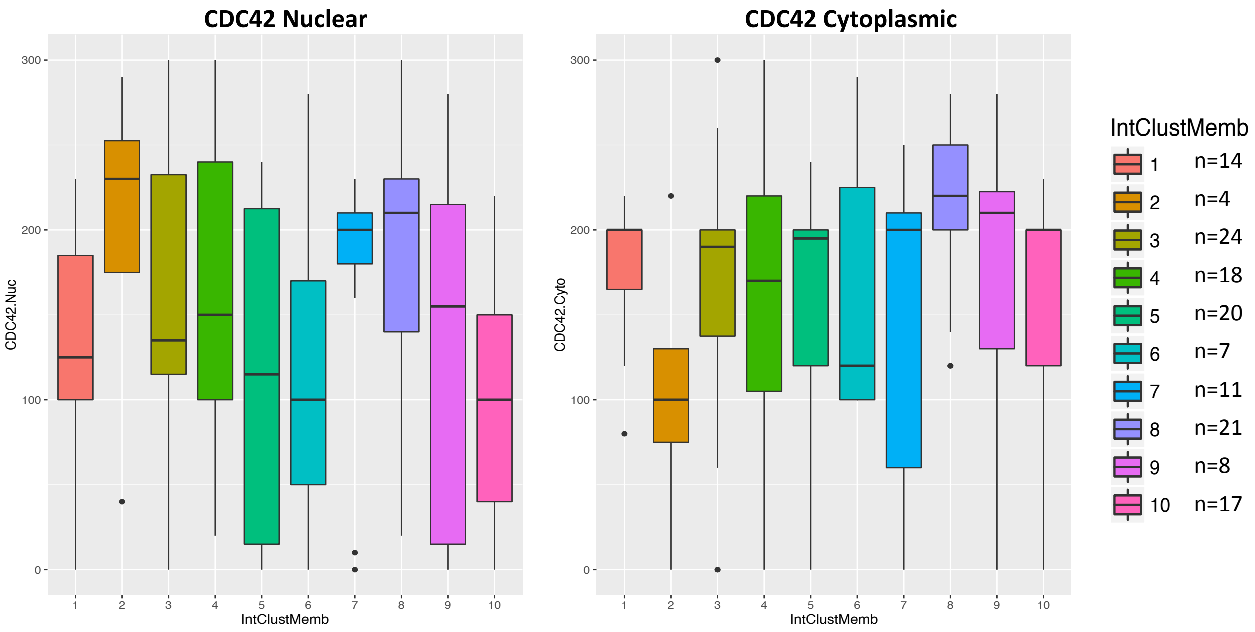

Distribution of CDC42 H-scores by Integrated Cluster membership of cases within METABRIC cohort.
